# Acupuncture treatment of Satoyoshi syndrome: a case report of a rare disease

**DOI:** 10.3389/fendo.2025.1543991

**Published:** 2025-05-29

**Authors:** Yu Gong, Xudong Zhang, Xiaomin Hao, Jipeng Liu, Bingnan Yue, Chuan Liu, Tianqi Xia, Yi Yang, Longteng Tu, WeiJie Qiu, Yu Liu, Qingguo Liu

**Affiliations:** ^1^ School of Acupuncture-Moxibustion and Tuina, Beijing University of Chinese Medicine, Beijing, China; ^2^ Department of Chinese Medicine, Beijing Jishuitan Hospital, Beijing, China; ^3^ Dongzhimen Hospital, Beijing University of Chinese Medicine, Beijing, China; ^4^ Department of Acupuncture and Moxibustion, Beijing Chaoyang District Wangjing Community Health Service Center, Beijing, China; ^5^ School of Special Education, Beijing Union University, Beijing, China

**Keywords:** acupuncture, Satoyoshi syndrome, case report, rare disease, autoimmune disorder

## Abstract

**Background:**

Satoyoshi syndrome (also known as Komuragaeri disease ) is a rare disorder of unknown etiology, with progressive muscle spasms, whole-body hair loss, and diarrhea as its main symptoms, particularly progressive skeletal muscle spasms and pain. Because of the lack of a clear etiology and pathogenesis of Satoyoshi syndrome, Western medicine lacks established effective therapies, and the long-term prognosis of the treatment of this disease is poor, unable to improve multiple symptoms simultaneously and prevent the recurrence of the disease. In recent years, acupuncture has been increasingly explored as a complementary treatment for autoimmune diseases. It is believed to exert its effects by modulating the neuroendocrine-immune network, enhancing immune cell function, and restoring homeostatic pathways. These mechanisms enable acupuncture to provide immune modulation, ultimately achieving a holistic and bidirectional regulatory effect.

**Case description:**

We report the case of a 54-year-old male police officer who had Satoyoshi syndrome for more than five years. After six months of acupuncture treatment, the patient’s chronic diarrhea completely disappeared, and the occasional painful muscle cramps and insomnia significantly improved. After six months of follow-up, the patient’s condition was stable.

**Conclusion:**

In this study, we believe that acupuncture therapy is of great significance for the improvement of diarrhea, immediate and long-term analgesia, and stabilization of the Satoyoshi syndrome.

## 1 Background

Satoyoshi syndrome (Ss), also known as Komuragaeri disease, represents a rare autoimmune disorder first reported in 1967 by Eijiro Satoyoshi and Kaneo Yamada. It is characterized by progressive, intermittent myalgia with distinctive multisystem manifestations (1, 2). The initial case report detailed a complex clinical presentation involving concurrent neurological impairments (3), endocrine dysfunction (4), skeletal abnormalities (5),and gastrointestinal disturbances (1).One study suggests that seventy-seven patients with Satoyoshi syndrome were identified between 1967 and 2021. The incidence is low, most commonly in young women (6), the onset is usually younger than 20 years of age, and recovery is poor (7). There is no gold standard for the diagnosis of Satoyoshi syndrome, which commonly presents as painful muscle cramps, hair loss throughout the body, chronic diarrhea (6), amenorrhea, and skeletal deformities in women (5, 8). According to the available literature, current treatment methods mainly include corticosteroids (9), immunosuppressants (10), muscle relaxants (11), and anticonvulsants (9). However, the effects of these drugs are limited and can only improve the symptoms of patients with shortcomings, such as dependence and adverse drug reactions (12, 13). Therefore, there is an urgent need for safe, effective, and convenient nondrug therapies to improve treatment.

Acupuncture has been used for thousands of years, spreading to Europe and the United States from the 16th to 19th centuries (14). In recent years, its clinical application in managing autoimmune diseases remains in the exploratory phase (15). Existing studies suggest that acupuncture, as a complementary means of conventional treatment, is gradually being regarded as a complementary and alternative treatment option for autoimmune diseases, which can be characterized as a holistic and bi-directional regulation by activating the human body’s immune cells, thereby stimulating the body’s intrinsic regulatory system (16).

While some studies have reported positive outcomes, the evidence remains limited. The mechanisms of action and therapeutic value are still debated in the academic community, and no clear or unified conclusion has yet been reached. Modern scientific research suggests that acupuncture primarily maintains human homeostasis by regulating the neuroendocrine-immune network (17–19). Furthermore, in terms of immune regulation, some studies hypothesize that acupuncture may exert an immune-modulating effect by enhancing the functions of NK and CD8+ T cells, as well as restoring the balance of Th1/Th2, Th17/Treg, and M1/M2 pathways (17). Evidence shows that acupuncture has effects in the treatment of multiple sclerosis (MS) (20), rheumatoid arthritis (RA) (21), and other immune diseases (22, 23) however, no studies have been conducted on the treatment of this disease using traditional Chinese medicine. Here, we report a case of Satoyoshi syndrome in which acupuncture improved painful muscle spasms, chronic diarrhea, and other symptoms.

## 2 Case presentation

A 54-year-old male police officer from China has been diagnosed with Satoyoshi syndrome for more than five years, and in early June 2023, the patient was admitted to the hospital due to a paroxysmal muscle spasm. The exact cause of Satoyoshi syndrome is unknown, but it is believed to have an autoimmune component. Occupational exposure analysis indicated that the patient was chronically high-stress (working an average of 60 hours/week and frequent night shifts for 12 years), which may contribute to immune dysregulation. The patient denied smoking or recreational drug use, but reported moderate alcohol consumption (20 g/day) prior to diagnosis and stopped drinking alcohol after diagnosis in 2018. It is hypothesized that Satoyoshi syndrome has an autoimmune origin, but its exact etiology remains unclear. In this case, occupational stress and circadian rhythm disruption have been suggested as potential moderators of neuroimmune axis dysfunction.

In this case, the patient presented with progressive limb muscle spasms, hair loss and chronic diarrhea, consistent with the core features of Satoyoshi syndrome. He was also differentiated from other diseases: Neurogenic muscle weakness syndrome was excluded because the patient tested negative for relevant muscle weakness-specific antibodies (anti-acetylcholine receptor antibody, anti-muscle-specific kinase antibody) were negative; autoimmune encephalitis was negative for oligoclonal bands in cerebrospinal fluid, and negative for anti-NMDAR and LGI1 antibodies; endocrine metabolic disorders were normal for thyroid function, cortisol, growth hormone and bone metabolism markers; hereditary myopathies were ruled out by whole-exome Genetic myopathies could be excluded by whole exome sequencing; tumor-associated myasthenia gravis was negative on whole body PET-CT and serum anti-Hu/Yo antibody screening.Combined with the significant reduction in the frequency of muscle spasms (symptoms initially suspected to be related to myasthenia gravis but later diagnosed as part of Satoyoshi syndrome) in response to oral immunosuppression, along with the secondary features of Satoyoshi syndrome such as reduced bone mineral density at follow - up, the diagnosis of Satoyoshi syndrome was confirmed.Treatment focuses on managing symptoms and may include medications to reduce muscle spasms and address other specific symptoms.

**Figure 1:**
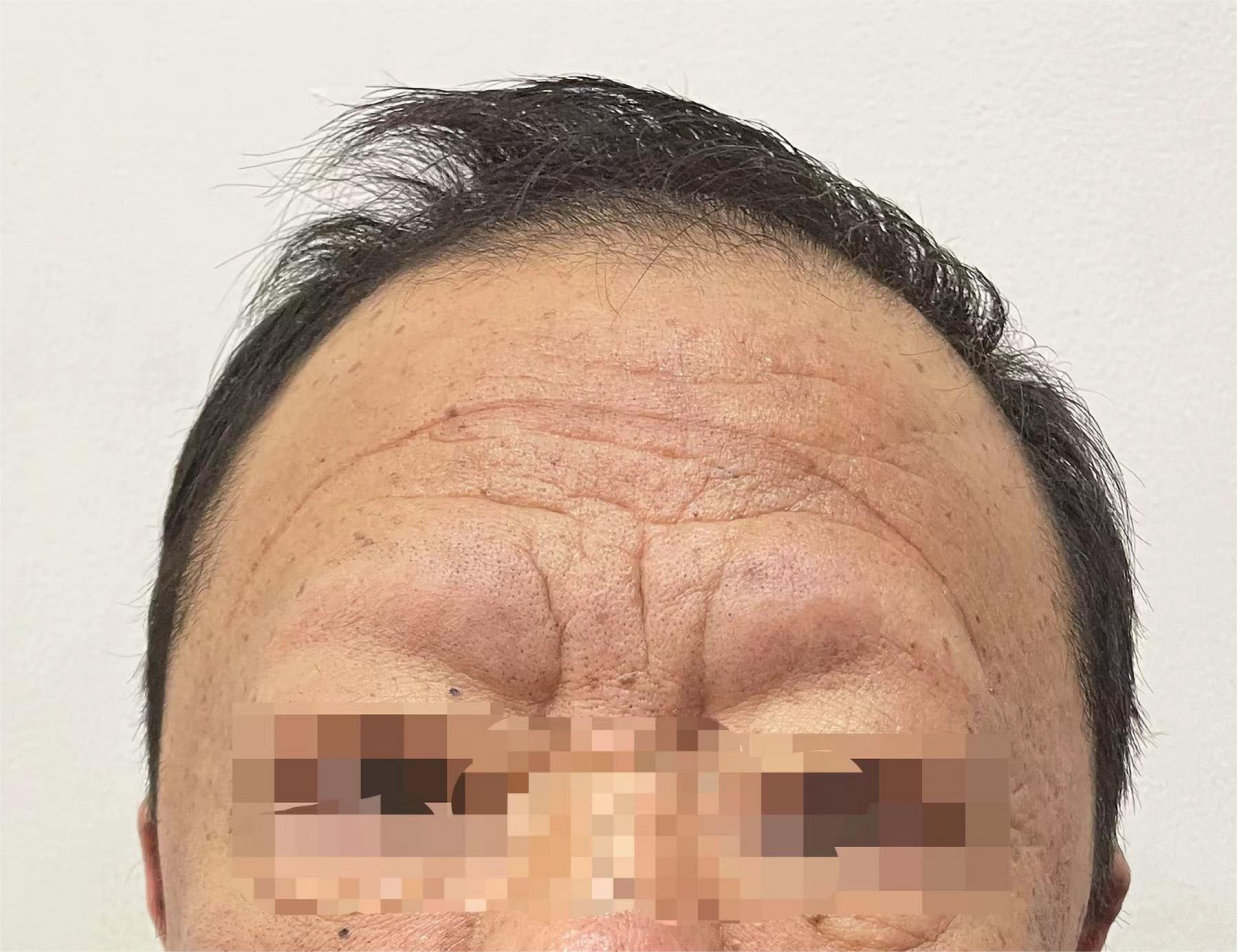
Patient’s eyebrow and hair loss diagram.

The patient’s symptoms began 12 years ago, with unexplained back spasms and pain that lasted approximately 10–20 minutes before disappearance. Since then, the symptoms have repeated several times a month or even several times a day. The patient recalled that since 2019, both upper limbs and lower legs began to have spasms, pain, and stiffness at the same time, which occurred more than 30 times a day, each time lasting approximately 10 minutes to relieve, and the nature of the pain was worse than before. It is worth noting that the patient gradually experienced hair loss in several locations, including the eyebrows, eyelashes, and hair, as shown in **Figure 1**. In addition, the patient’s face loses its usual luster, and the skin darkens. Since the onset of and repeated diarrhea,(characterized by loose, soft, and shapeless stool occurring 4–6 times a day), there was no significant change in weight. Dysphagia, dyspnea, chest tightness, palpitations, and other symptoms were absent.

At admission, physical examination: the patient’s vital signs were normal (T 36.2°C, P 60 beats/min, R 18 breaths/min, BP 120/78 mmHg), and cardiopulmonary examination showed no abnormality. Skin examination showed that the face had lost its luster, hair in the eyebrows had fallen out, and the hair quality was sparse and soft. A neurological examination revealed that the patient was conscious and fluent in speech. Abdominal wall reflexes were present. The proximal and distal muscle strength of both upper limbs was grade V,suggesting that the patient did not have significant myasthenia gravis or motor neuron injury. The proximal and distal muscle strength of both lower limbs was also grade V. The biceps, triceps, and membrane reflexes of both upper limbs were symmetrical (+). The knee and Achilles tendon reflexes of both lower limbs were symmetrical (+), suggesting an intact spinal reflex arc. No other abnormalities were found during the clinical examination.

Routine blood results showed eosinophilia, which is common in autoimmune diseases and allergic reactions. potassium (3.45 mmol/L) and hemoglobin A1c levels were normal.

Immunohistochemical results revealed a flow cytometry of CD20 (a few positive lymphocytes) and CD3 (some positive lymphocytes), suggesting an immune response with synergistic involvement of B-T cells (8). Serum immunofixation electrophoresis showed increased monoclonal immunoglobulin IgG, suggesting clonal proliferation of plasma cells (9). κlight chain, and β2 microglobulin 2.02 (mg/L), whose elevation reflects lymphocyte activation or inflammatory state and correlates with autoimmune disease activity. M (7.9%) and light chain proteins in hematuria were normal,exclude plasma cell malignancies such as multiple myeloma and support the nonmalignant immunodysregulatory nature of Satoyoshi’s syndrome. Antibrain tissue, antiglutamate decarboxylase, and antinuclear antibodies were not detected.

Whole-body bone imaging showed a concentration of radioactivity in the upper and lower mandibles and the left sacroiliac joint, and the radioactivity in the left upper bone was reduced, It may reflect abnormal bone metabolism or localized inflammation, which correlates with skeletal malformations (e.g., premature epiphyseal closure) reported in the literature for Satoyoshi syndrome (24).

**Figure 2:**
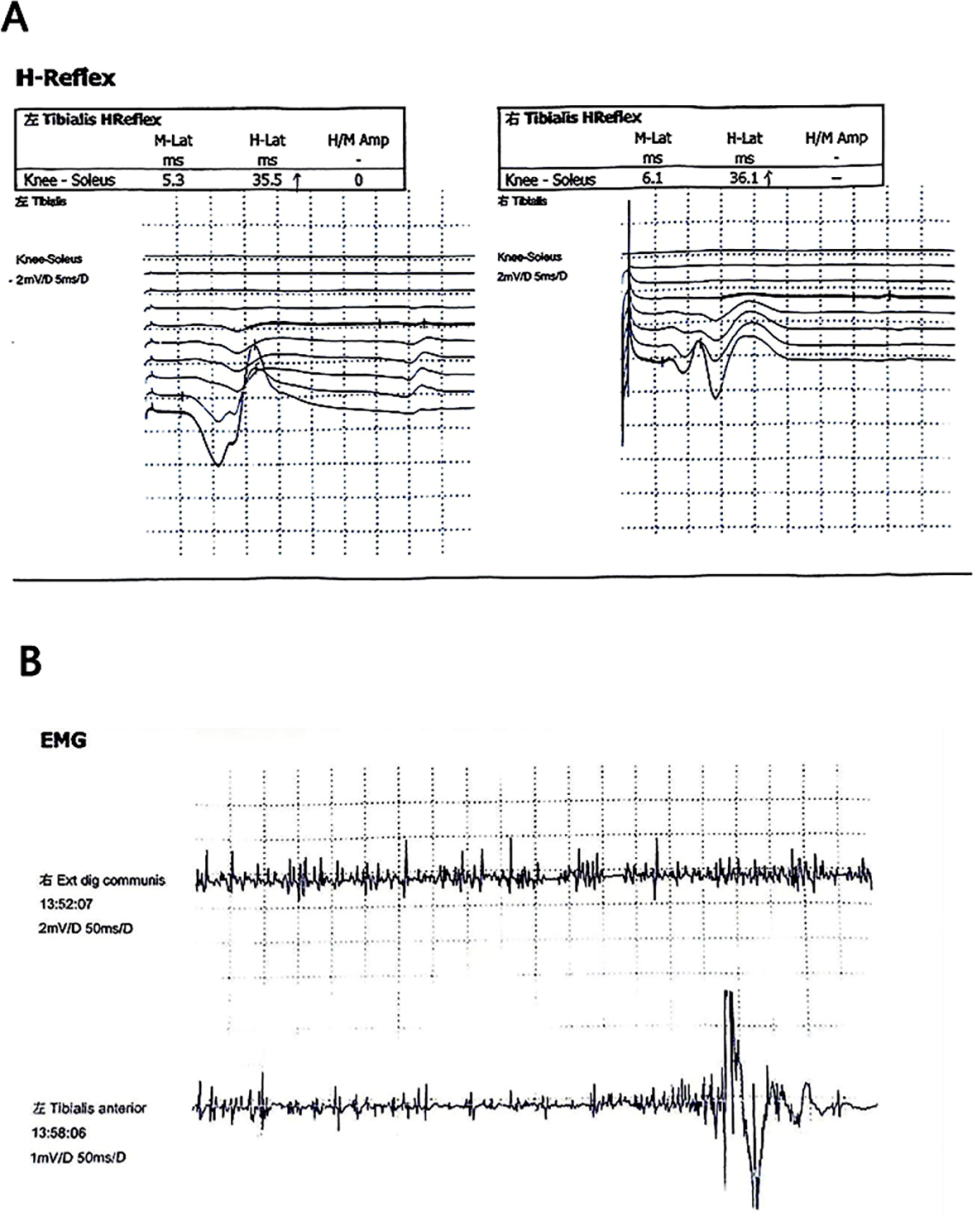
**(A)** The latency of bilateral tibial nerve H reflex is prolonged. **(B)** Neuromuscular electrogram (EMG) suggested that there was strong electrical activity in the left tibialis anterior.

Neuroimaging indicated prolonged latency of the bilateral tibial nerve H-reflex,suggesting decreased excitability of spinal alpha motor neurons (25). Neuromuscular electrogram (EMG) suggested that there was strong electrical activity in the left tibialis anterior. For details, see **Figures 2A**, **B**. The muscle biopsy Pathological examination of the biopsy tissues revealed mild myogenic lesions, mild neurogenic atrophy, myofibrillar destruction, and a slight increase in mitochondria. In addition, bone marrow biopsy results suggested eosinophilic infiltration.

During hospitalization, the patient was treated with intravenous injections of rituximab (100 mg), carbamazepine (0.2 g, twice daily, and baclofen (10 mg, twice daily) (2, 4, 26, 27). Eleven days after hospitalization, the pain and spasmodic symptoms were relieved and the frequency of attacks was reduced. After discharge, the patient changed to oxamazepine (0.3 g, twice daily owing to self-reported memory loss. The patient continued to take baclofen (10 mg twice daily) to alleviate the painful cramps. One week prior to the patient visiting a Traditional Chinese Medicine (TCM) Clinic on July 4, 2023, The patient experienced severe pain and severe contraction of the lower extremities, accompanied by insomnia, diarrhea, elevated blood pressure, and other symptoms. Despite having stopped drinking alcohol in 2018, the patient had recently relapsed. Given the patient’s pre-existing Satoyoshi syndrome, the frequent alcohol consumption likely exacerbated these symptoms. Alcohol can interfere with muscle function, irritate the gastrointestinal tract, and affect blood pressure, which may have contributed to the observed exacerbation of his condition.

After the patient’s first visit to the clinic, an experienced acupuncturist performed a detailed TCM diagnosis and found that the patient had a dull complexion and loss of facial eyebrows. The patient self-reported muscle aches and pain in his limbs, especially after exertion and in rainy weather, accompanied by a feeling of muscle stiffness. His diet was fine, but she had loose stools 4-6 times a day. Her tongue was pale and fat, with teeth marks on the sides, white and greasy moss, and moist and slow pulse. After our detailed traditional Chinese medicine(TCM) dialectical diagnosis, we found that this disease belongs to the syndrome of “myobi” described in the ancient TCM book “Huangdi Neijing”.At the same time, the acupuncturist made a treatment plan for the patient and instructed the patient to undergo acupuncture twice a week for three months. Oxamazepine and baclofen were continued to stabilize the patient during the treatment period. In acupuncture treatment, the procedure was performed by a licensed acupuncturist with at least 5 years of professional qualification. The acupuncturist determined the acupoints in strict accordance with the National Standard for Acupoint Positioning.

**Figure 3:**
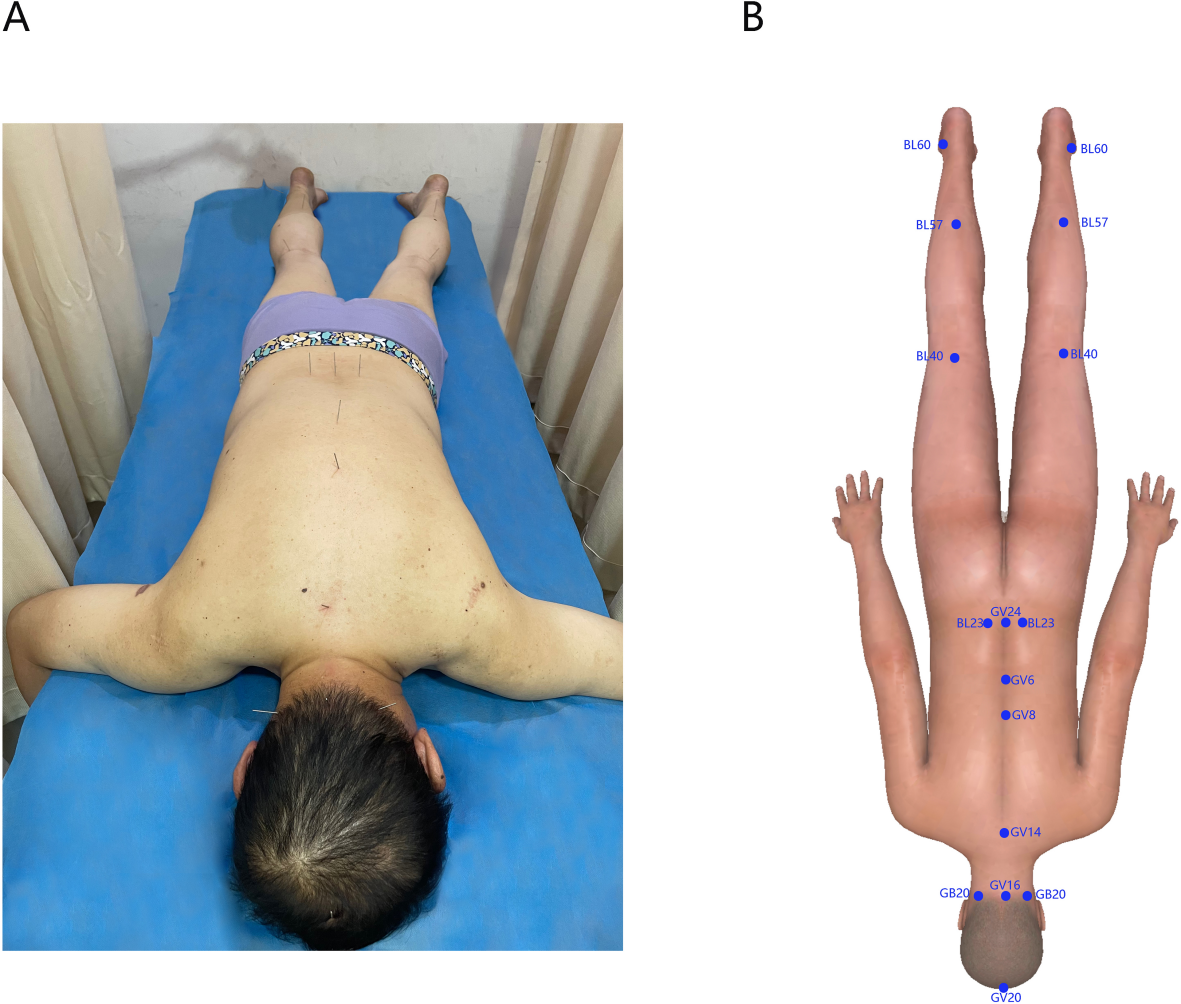
**(A)** Photos of patients undergoing acupuncture treatment. **(B)** Software generated 3D-body image showing the selected acupoints for treatment.

Disposable sterile stainless- steel acupuncture needles of the Hanyi brand (specification: 0.35 mm×40 mm) were used. These acupoints included Baihui (GV20), Fengchi (GB20), Fengfu (GV16), Dazhui (GV14), Geshu (BL17), Jinsuo (GV8), Mingmen (GV4), Shenshu (BL23), Weizhong (BL40), Chengshan (BL57), and Kunlun (BL60), specific acupoints are shown in **Figures 3A**, **B**.

**Figure 4:**
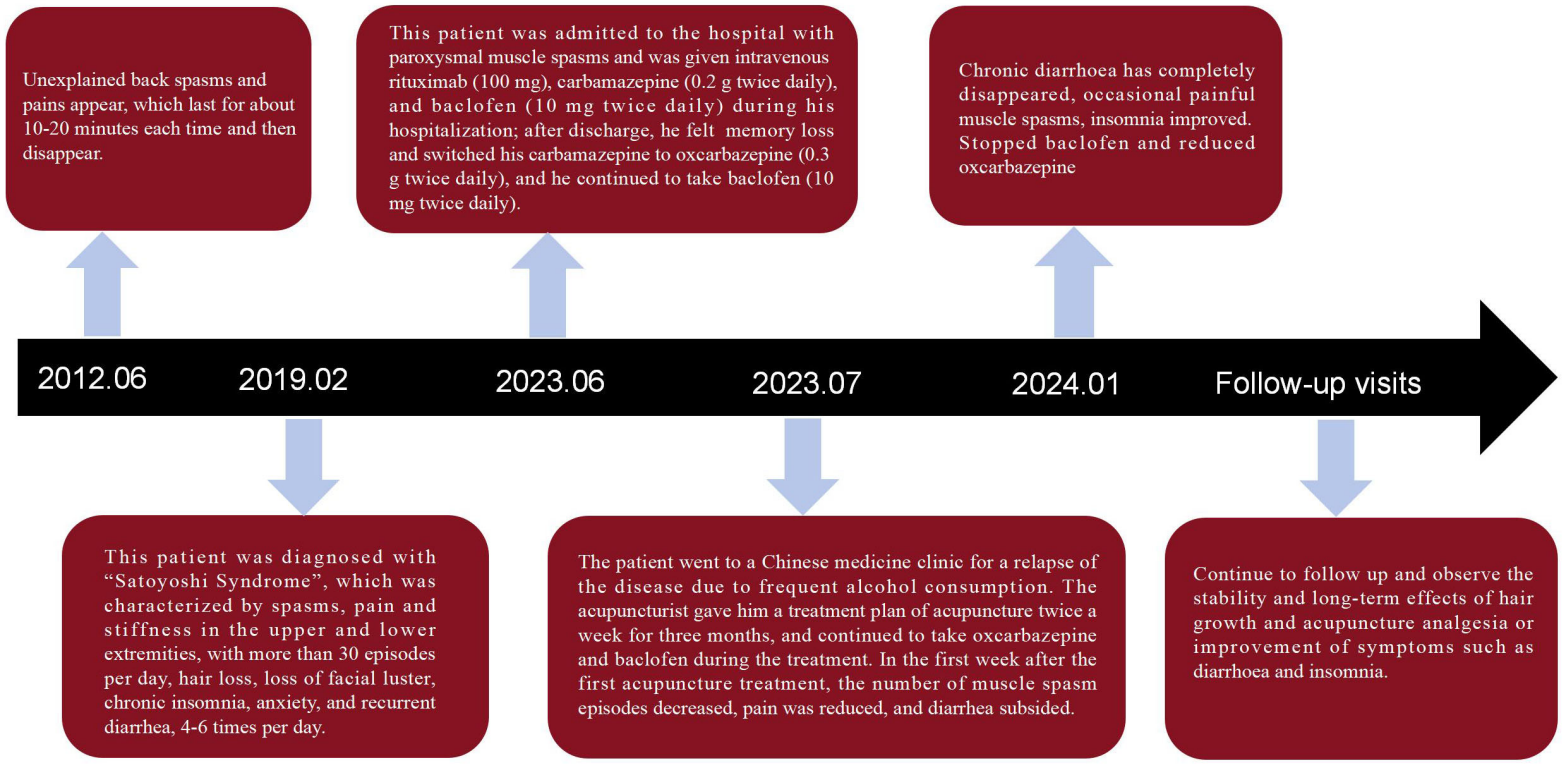
Flowchart of patient’s time to treatment.

**Figure 5:**
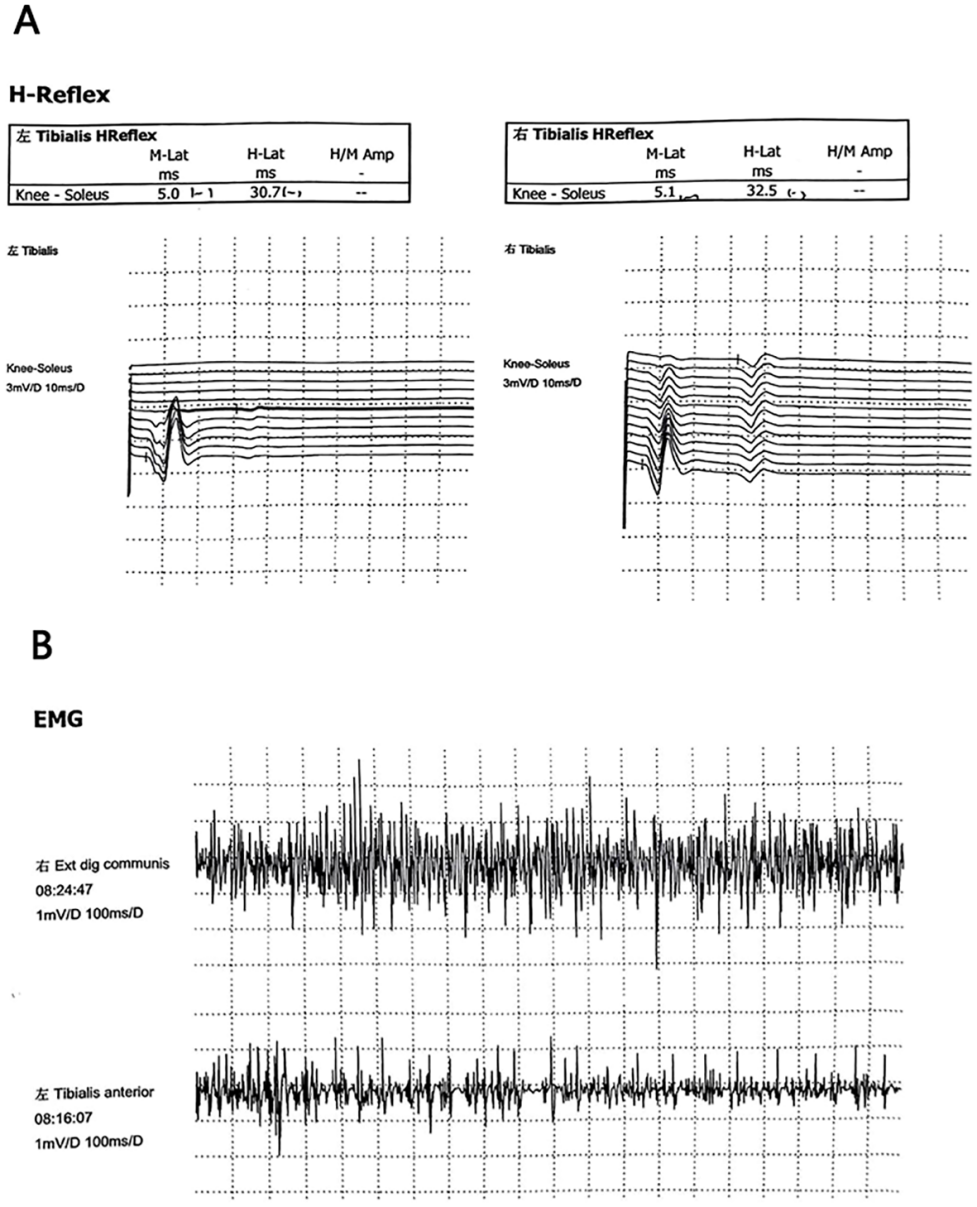
**(A)** H reflex of bilateral tibial nerve is normal. **(B)** Electromyography (EMG) showed that there was no abnormal discharge in the right extensor digitorum communis and the left tibialis anterior, and no neurogenic or myogenic damage was found.

The patient was placed in the prone position. The acupuncturist used a standardized technique of “twisting flat tonic and cathartic” to insert the needles. Each time, the needles were left in place for 30 minutes, and the treatment was administered 2 times a week for a total of 14 times. The entire treatment process was conducted in a quiet environment with a constant temperature of (25 ± 2)°C. After 14 acupuncture treatments, the patient’s diarrhea, muscle pain, and spasm episodes were significantly reduced, and sleep and mood improved; however, the hair shed did not grow temporarily. The patient was asked to discontinue baclofen and take oxcarbamazepine (0.3 g, once daily at a reduced dose. The patient was advised to continue treatment once a week for three months to stabilize his condition. In the week after the first acupuncture session, muscle spasms only occurred twice, pain relief was no longer necessary to rely on painkillers to alleviate the condition, and the frequency of loose stools was reduced to 1-3 times a day. After three months of continuous treatment, the chronic diarrhea had disappeared entirely, with occasional painful muscle cramps; another strange phenomenon is that the patient’s body weight also decreased by 5 kg. After six months of follow-up, the patient’s condition stabilized, and no adverse events were observed. The timelines of the interventions and specific treatment effects are shown in **Figure 4**. Patients were asked to review regularly. The results of a review in April 2024 indicated that there were no apparent abnormalities in routine blood, urine, stool, liver, or kidney function. Neuromuscular electrogram (EMG) suggested that the amplitude in both lower limbs was normal, with reasonable repeatability and a normal incubation period. No neurogenic or myogenic damage was observed in the patient’s neuromuscular limbs. Neuroimaging showed that the bilateral tibial nerve H reflex was normal.For details, see **Figures 5A**, **B**. M protein (6.5%). The bone marrow cell morphology was not abnormal. Flow cytometry analysis revealed the proportion of mature cells.

## 3 Discussion

Acupuncture is an important component of traditional Chinese medicine. In recent years, acupuncture has become increasingly popular worldwide in recent years (28, 29). This case report is the first to describe the use of acupuncture for the treatment of Satoyoshi syndrome, with impressive results. The patient’s painful muscle spasms were not well controlled by traditional analgesics during previous treatment. However, our patient successfully relieved the pain symptoms after the first acupuncture treatment. Subsequently, we observed a significant reduction in the number of episodes of muscle spasms, the disappearance of diarrhea, and the improvement or disappearance of many symptoms, such as muscle rigidity. During the follow-up period of 6 months, the patient’s condition remained stable. There is no doubt that our study preliminarily confirmed the immediate and long-term therapeutic effect of acupuncture on this disease.

A previous review showed a significant association between Satoyoshi syndrome and autoimmune factors (7). Immunological tests have shown that patients are usually positive for serum antinuclear antibodies (30, 31), anti-acetylcholine receptor antibodies (32), glutamate decarboxylase antibodies (33), and anti-DNA antibodies (12). Other studies have shown a tendency towards higher levels of immunoglobulin IgG (9), IgE (34), IgM (32), or anti-olysin antibodies (35). IgG antibodies were detected in the serum of patients; therefore, it is presumed that an immune disorder is the leading cause of the disease. Histopathological examination of the gastrointestinal tract has revealed inflammatory lymphocyte infiltration (8), and histopathological studies of scalp biopsy specimens have shown similar results (9). The exact cause of Satoyoshi syndrome is unknown, although autoimmune factors have been speculated to be the likely cause. Recent studies have confirmed that neuromyography is an essential tool for detecting the cause of muscle contractions (36). Our patient was primarily affected by involuntary muscle contractions. Therefore, we performed neuromyography to clarify the electrophysiological characteristics of the disease. Evidence from animal experiments and clinical trials shows that acupuncture as a safe and effective nondrug therapy for the regulation of the immune system has gradually been widely recognized, including allergic diseases (37), autoimmune diseases (23), and immune deficiency syndrome (38). For example, acupuncture at Zusanli can increase the gene content of tissue repair growth factor and the gene expression of anti-inflammatory cytokines and mobilize the immune response mechanism to promote tissue healing in rheumatoid arthritis (RA) (39). In addition, we found that acupuncture could prevent suppurative organ damage by inhibiting excessive inflammatory responses and resisting oxidative stress, improving the immunosuppressive state of patients with sepsis, and maintaining immune homeostasis (40).

Several other systems, including the nervous, gastrointestinal, and skin systems, were involved, in addition to the immune system. After the initial treatment, the severity of pain and the number of seizures were significantly reduced. Clinical studies have shown that acupuncture is effective in relieving the pain associated with multiple sclerosis (MS), reducing extremity spasticity, and restoring mobility (20). We believe that the improvement in neurological symptoms in these patients may be related to the anti-inflammatory and analgesic effects of acupuncture.

Surprisingly, after six months of treatment, the patient’s diarrhea completely disappeared. A recent study showed that acupuncture can regulate the expression of AQP1, AQP3, AQP8, and TJs, restoring water metabolism and intestinal permeability balance in IBS-D to relieve diarrhea symptoms in rats (41). A systematic review also showed that acupuncture therapy significantly reduced the number of stools per week in patients with IBS-D (42). More importantly, this is consistent with the “holistic concept” of traditional Chinese medicine, in which acupuncture can regulate the imbalance of the nervous, gastrointestinal, and skin systems caused by the disease and improve the overall symptoms of patients.

When treating this patient, we chose acupuncture points on “Governor Vessel (GV)” and “Bladder Meridian of Foot-Taiyang (BL)” and took “dispelling wind and dispelling evil, resolving muscle and clearing collaterals” as the treatment principle. Previous studies have shown that acupuncture at the Baihui, Fengchi, and Dazhui points can be widely used for the treatment of nervous system or immune diseases (21, 37, 43, 44). Multiple studies have confirmed that acupuncture at the Weizhong, Chengshan, and Kunlun points can reduce the pain threshold and effectively relieve chronic pain in the lower limbs (45–47).

In this case, medication adjustments (especially oxcarbazepine dose changes with baclofen discontinuation) and alcohol consumption were the main confounders, which may have bi-directionally interfered with the assessment of acupuncture efficacy. In this case, wet weather (the patient reported worsening of symptoms on rainy days) may have exacerbated the muscle pain; alcohol may increase the release of inflammatory factors and exacerbate muscle spasm.

However, owing to the lack of case reports and randomized controlled trials on acupuncture in the treatment of Satoyoshi syndrome, we cannot speculate on the specific mechanism of action of acupuncture in the treatment of this disease. In the future, we will pay close attention to hair growth in patients; therefore, more clinical evidence or Long-term clinical efficacy and safety of this treatment.

## 4 Conclusion

In conclusion, our case report demonstrates that acupuncture, as a non-pharmacological treatment can significantly alleviate a wide range of symptoms, including muscle spasms, chronic diarrhea, and insomnia, while reducing the dosage of Western medications. Notably, acupuncture not only mitigated adverse reactions to Western drugs but also compensated for their single-target effects. For rare diseases like Satoyoshi syndrome, which affects the neuro - digestive-immune system and lacks effective Western medical treatments, acupuncture can modulate the neuroendocrine - immune network. This modulation improves multi - system symptoms and lessens patients’ dependence on medications such as baclofen and oxcarbazepine. Therefore, clinicians should consider incorporate acupuncture as an adjunctive therapy in the treatment of rare diseases, refractory diseases, and diseases involving multiple systems. For patients with chronic immune diseases who experience adverse drug reactions, acupuncture not only optimizes the safety of treatment but also exerts holistic regulatory effects, thereby enhancing overall therapeutic outcomes.

Additionally, acupuncture can be explored as an adjunctive intervention to a comprehensive treatment strategy for immune system disorders, offering a complementary option to the current standard regimen for Satoyoshi syndrome. This approach may improve overall outcomes and prognosis for patients with immune disorders. Finally, it is worth noting that we need to carry out long-term follow-up monitoring of patients, extend the follow-up period, and regularly recommend patients to undergo comprehensive examinations(e.g., neuromuscular electromyography) and assessments. Through long-term follow-up, observe the changes of disease symptoms, monitor disease recurrence, and assess the long-term efficacy and safety of acupuncture treatment. Data on patients’ functional status in daily life, such as mobility and self-care ability, are collected to reflect more comprehensively the long-term impact of acupuncture treatment on patients’ quality of life.We believe that acupuncture therapy is of great significance for immediate and long-term analgesia and improvement of diarrhea and stability in patients with Satoyoshi syndrome. However, further clinical evidence is required to confirm the mechanism and efficacy of acupuncture.

## Data Availability

The raw data supporting the conclusions of this article will be made available by the authors, without undue reservation.

## References

[1] Solís-García Del Pozo J de Cabo C Solera J . Treatment of Satoyoshi syndrome: a systematic review. Orphanet J Rare Dis. (2019) 14:146. doi: 10.1186/s13023-019-1120-7 31217029 PMC6585110

[2] Satoyoshi E Yamada K . Recurrent muscle spasms of central origin. A Rep two cases. Arch Neurol. (1967) 16:254–64. doi: 10.1001/archneur.1967.00470210030004 6018875

[3] Merello M García H Nogués M Leiguarda R . Masticatory muscle spasm in a non-Japanese patient with Satoyoshi syndrome successfully treated with botulinum toxin. Mov Disord. (1994) 9:104–5. doi: 10.1002/mds.870090118 8139588

[4] Heger S Kuester RM Volk R Stephani U Sippell WG . Satoyoshi syndrome: a rare multisystemic disorder requiring systemic and symptomatic treatment. Brain Dev. (2006) 28:300–4. doi: 10.1016/j.braindev.2005.10.006 16478652

[5] Haymon M Willis RB Ehlayel MS Lacassie Y . Radiological and orthopedic abnormalities in Satoyoshi syndrome. Pediatr Radiol. (1997) 27:415–8. doi: 10.1007/s002470050158 9133353

[6] Ikeda K Satoyoshi E Kinoshita M Wakata N Iwasaki Y . Satoyoshi’s syndrome in an adult: a review of the literature of adult onset cases. Intern Med. (1998) 37:784–7. doi: 10.2169/internalmedicine.37.784 9804090

[7] Viana Abreu Montanaro V Solís-García Del Pozo J Falcão Hora T León BH de Cabo C Solera J . Is Satoyoshi syndrome an autoimmune disease? A systematic review. Rheumatol (Oxf Engl). (2023) 62:2343–51. doi: 10.1093/rheumatology/kead067 36749015

[8] Solís-García Del Pozo J de Cabo C Solera J . Gastrointestinal manifestations in Satoyoshi syndrome: a systematic review. Orphanet J Rare Dis. (2020) 15:115. doi: 10.1186/s13023-020-01395-8 32429959 PMC7236136

[9] Wisuthsarewong W Likitmaskul S Manonukul J . Satoyoshi syndrome. Pediatr Dermatol. (2001) 18:406–10. doi: 10.1046/j.1525-1470.2001.01966.x 11737686

[10] Montanaro VVA Hora TF Couto CM Ribas FD . Adult-onset Satoyoshi syndrome in a young male. Neuromuscul Disord. (2017) 27:382–4. doi: 10.1016/j.nmd.2017.01.007 28215594

[11] Mukhopadhyay D Ghosh A Mukhopadhyay M . Satoyoshi syndrome. Indian Pediatr. (2011) 48:729–31. 21992906

[12] Arita J Hamano S Nara T Maekawa K . Intravenous gammaglobulin therapy of Satoyoshi syndrome. Brain Dev. (1996) 18:409–11. doi: 10.1016/0387-7604(96)00033-2 8891238

[13] Ishihara M Ogawa K Suzuki Y Kamei S Ochiai T Sonoo M . Adult-onset Satoyoshi syndrome with prominent laterality of clinical features. Intern Med. (2014) 53:2811–6. doi: 10.2169/internalmedicine.53.2958 25500444

[14] Mallory MJ Do A Bublitz SE Veleber SJ Bauer BA Bhagra A . Puncturing the myths of acupuncture. J Integr Med. (2016) 14:311–4. doi: 10.1016/S2095-4964(16)60269-8 27641603

[15] Couillard F Parreau S Dumonteil S Ratti N Palat S Bezanahary H . Use of complementary and alternative medicine by patients treated for systemic lupus erythematosus, primary sjögren’s syndrome, or systemic sclerosis in a french rural region. Complement Med Res. (2024) 31:234–40. doi: 10.1159/000536580 38346411

[16] Schwartz N Chalasani MLS Li TM Feng Z Shipman WD Lu TT . Lymphatic function in autoimmune diseases. Front Immunol. (2019) 10:519. doi: 10.3389/fimmu.2019.00519 30949174 PMC6435962

[17] Liu F Wang Y Lyu K Du X Zhou M Shi J . Acupuncture and its ability to restore and maintain immune homeostasis. QJM. (2024) 117:167–76. doi: 10.1093/qjmed/hcad134 37318994

[18] Ding SS Hong SH Wang C Guo Y Wang ZK Xu Y . Acupuncture modulates the neuro-endocrine-immune network. QJM Mon J Assoc Phys. (2014) 107:341–5. doi: 10.1093/qjmed/hct196 24106314

[19] Wang N Zhao L Zhang D Kong F . Research progress on the immunomodulatory mechanism of acupuncture in tumor immune microenvironment. Front Immunol. (2023) 14:1092402. doi: 10.3389/fimmu.2023.1092402 36865562 PMC9971227

[20] Khodaie F Abbasi N Kazemi Motlagh AH Zhao B Naser Moghadasi A . Acupuncture for multiple sclerosis: A literature review. Mult Scler Relat Disord. (2022) 60:103715. doi: 10.1016/j.msard.2022.103715 35259684

[21] Xu Y Hong S Zhao X Wang S Xu Z Ding S . Acupuncture alleviates rheumatoid arthritis by immune-network modulation. Am J Chin Med. (2018) 46:997–1019. doi: 10.1142/S0192415X18500520 30001644

[22] Wang H Yang G Wang S Zheng X Zhang W Li Y . The most commonly treated acupuncture indications in the United States: A cross-sectional study. Am J Chin Med. (2018), 1–33. doi: 10.1142/S0192415X18500738 30298749

[23] Wang J Zhu F Huang W Chen Z Zhao P Lei Y . Therapeutic effect and mechanism of acupuncture in autoimmune diseases. Am J Chin Med. (2022) 50:639–52. doi: 10.1142/S0192415X22500252 35282807

[24] Venegas-Vega CA Rivera-Vega MR Cuevas-Covarrubias S Orozco J Kofman-Alfaro S . Satoyoshi syndrome with unusual skeletal abnormalities and parental consanguinity. Am J Med Genet A. (2009) 149A:2448–51. doi: 10.1002/ajmg.a.32751 19839037

[25] Pardal-Fernández JM Solera-Santos J Iniesta-López I Rodríguez-Vázquez M . Satoyoshi’s syndrome related muscle spasms: functional study. Rev Neurol (Paris). (2012) 168:291–5. doi: 10.1016/j.neurol.2011.06.004 22100320

[26] Li J Peng D Jiang T Avivi-Arber L . Satoyoshi syndrome with progressive orofacial manifestations: A case history report. Int J Prosthodont. (2017) 30:163–7. doi: 10.11607/ijp.4905 28267828

[27] See S Ginzburg R . Skeletal muscle relaxants. Pharmacotherapy. (2008) 28:207–13. doi: 10.1592/phco.28.2.207 18225966

[28] Patil S Sen S Bral M Reddy S Bradley KK Cornett EM . The role of acupuncture in pain management. Curr Pain Headache Rep. (2016) 20:22. doi: 10.1007/s11916-016-0552-1 26896946

[29] Kaptchuk TJ . Acupuncture: theory, efficacy, and practice. Ann Intern Med. (2002) 136:374–83. doi: 10.7326/0003-4819-136-5-200203050-00010 11874310

[30] Mani V George R . Satoyoshi syndrome-A case report from India. Pediatr Dermatol. (2017) 34:e296–8. doi: 10.1111/pde.13271 28940615

[31] Rudnicka L Kwiatkowska M Rakowska A Czuwara J Olszewska M . Alopecia areata. How not to miss Satoyoshi syndrome? J Dermatol. (2014) 41:951–6. doi: 10.1111/1346-8138.12633 25289915

[32] Endo K Yamamoto T Nakamura K Hoshi A Yamanoi T Watanabe A . Improvement of Satoyoshi syndrome with tacrolimus and corticosteroids. Neurology. (2003) 60:2014–5. doi: 10.1212/01.wnl.0000067994.01098.a5 12821760

[33] Drost G Verrips A Hooijkaas H Zwarts M . Glutamic acid decarboxylase antibodies in Satoyoshi syndrome. Ann Neurol. (2004) 55:450–1. doi: 10.1002/ana.20007 14991831

[34] Matsuura E Matsuyama W Sameshima T Arimura K . Satoyoshi syndrome has antibody against brain and gastrointestinal tissue. Muscle Nerve. (2007) 36:400–3. doi: 10.1002/mus.20773 17405137

[35] Merino de Paz N Rodriguez-Martin M Contreras Ferrer P Eliche MP Noda Cabrera A . Satoyoshi syndrome: a cause of alopecia universalis in association with neurologic and bony abnormalities. Pediatr Dermatol. (2013) 30:e22–4. doi: 10.1111/j.1525-1470.2012.01773.x 22612551

[36] Drost G Verrips A van Engelen BGM Stegeman DF Zwarts MJ . Involuntary painful muscle contractions in Satoyoshi syndrome: a surface electromyographic study. Mov Disord. (2006) 21:2015–8. doi: 10.1002/mds.21088 16972238

[37] Zheng H Xiao XJ Shi YZ Zhang LX Cao W Zheng QH . Efficacy of acupuncture for chronic spontaneous urticaria: A randomized controlled trial. Ann Intern Med. (2023) 176:1617–24. doi: 10.7326/M23-1043 37956431

[38] Duan Y Xu Z Deng J Lin Y Zheng Y Chen J . A scoping review of cohort studies assessing traditional Chinese medicine interventions. BMC Complement Med Ther. (2020) 20:361. doi: 10.1186/s12906-020-03150-9 33228628 PMC7684743

[39] Dong ZQ Zhu J DZ Lu Chen Q Xu YL . Effect of electroacupuncture in ‘Zusanli’ and ‘Kunlun’ Acupoints on TLR4 signaling pathway of adjuvant arthritis rats. Am J Ther. (2018) 25:e314–9. doi: 10.1097/MJT.0000000000000477 27574922

[40] Yang L Zhou D Cao J Shi F Zeng J Zhang S . Revealing the biological mechanism of acupuncture in alleviating excessive inflammatory responses and organ damage in sepsis: a systematic review. Front Immunol. (2023) 14:1242640. doi: 10.3389/fimmu.2023.1242640 37753078 PMC10518388

[41] Kang X Zhang H Li X Zhang K Huang Z Li Y . Electroacupuncture improving intestinal barrier function in rats with irritable bowel syndrome through regulating aquaporins. Dig Dis Sci. (2024) 69:1143–55. doi: 10.1007/s10620-024-08288-x 38421507

[42] Guo J Xing X Wu J Zhang H Yun Y Qin Z . Acupuncture for adults with diarrhea-predominant irritable bowel syndrome or functional diarrhea: A systematic review and meta-analysis. Neural Plast. (2020) 2020:8892184. doi: 10.1155/2020/8892184 33299403 PMC7705439

[43] Zhou M Pang F Liao D He X Yang Y Tang C . Electroacupuncture at Fengchi(GB20) and Yanglingquan(GB34) ameliorates paralgesia through microglia-mediated neuroinflammation in a rat model of migraine. Brain Sci. (2023) 13:541. doi: 10.3390/brainsci13040541 37190506 PMC10136420

[44] Zhao H Dong F Li Y Ren X Xia Z Wang Y . Inhibiting ATG5 mediated autophagy to regulate endoplasmic reticulum stress and CD4+ T lymphocyte differentiation: mechanisms of acupuncture’s effects on asthma. BioMed Pharmacother. (2021) 142:112045. doi: 10.1016/j.biopha.2021.112045 34426257

[45] Wei H Liu B Yin C Zeng D Nie H Li Y . Electroacupuncture improves gout arthritis pain via attenuating ROS-mediated NLRP3 inflammasome overactivation. Chin Medicine. (2023) 18:86. doi: 10.1186/s13020-023-00800-1 PMC10355064 37464384

[46] Jiang H Yu X Ren X Tu Y . Electroacupuncture alters pain-related behaviors and expression of spinal prostaglandin E2 in a rat model of neuropathic pain. J Tradit chin Med Chung Tsa Chih Ying wen pan. (2016) 36:85–91. doi: 10.1016/s0254-6272(16)30013-9 26946624

[47] Yang Y Wei X Tian J Zhu Y Jia S Shu Q . Scalp electroacupuncture targeting the VTADA neurons to relieve negative emotions and promote the alleviation of chronic pain. Front Neurosci. (2023) 17:1323727. doi: 10.3389/fnins.2023.1323727 38188034 PMC10771389

